# Supplementary material for: Abundant toxin-related genes in the genomes of beneficial symbionts from deep-sea hydrothermal vent mussels
Source: eLife. 2015 Sep 15;4:e07966. doi: 10.7554/eLife.07966 (PMC4612132; doi:10.7554/eLife.07966)
Supplement: Figure 6—source data 1. — DOI: http://dx.doi.org/10.7554/eLife.07966.021 [file elife07966s001.docx]

**Figure 6-Source data file.** **Variability in toxin-related genes encoded by the *Bathymodiolus* SOX symbionts**

| **ID** | **SNPs** | **Length** | **N’s** | **SNPs per gene** | **Annotation** | **Foreign** |
| --- | --- | --- | --- | --- | --- | --- |
| BAT00001 | 4 | 1413 | 947 | 8.6 | Hemolysin-type calcium binding protein | * |
| BAT00002 | 17 | 3741 | 2570 | 14.5 | RTX toxin protein | * |
| BAT00052-EXT | 54 | 2169 | 0 | 24.9 | RTX toxin | * |
| BAT00093 | 18 | 1029 | 0 | 17.5 | Insecticidal toxin complex-like protein |  |
| BAT00101 | 2 | 453 | 0 | 4.4 | Conserved hypothetical protein (RHS) |  |
| BAT00102 | 12 | 5193 | 3599 | 7.5 | Insecticidal toxin protein | * |
| BAT00152 | 11 | 2634 | 824 | 6.1 | [MARTX1] protein, filamentous hemagglutinin family |  |
| BAT00153 | 14 | 10764 | 4693 | 2.3 | [MARTX1] hemagglutination activity domain protein | * |
| BAT00154 | 33 | 4614 | 1458 | 10.5 | [MARTX1] hypothetical protein | * |
| BAT00155 | 0 | 9093 | 4373 | 0 | [MARTX1] hypothetical protein | * |
| BAT00156 | 0 | 4413 | 2859 | 0 | [MARTX1] hypothetical protein | * |
| BAT00157 | 0 | 5013 | 2894 | 0 | [MARTX1] hypothetical protein | * |
| BAT00158 | 10 | 4137 | 787 | 3 | [MARTX1] hypothetical protein | * |
| BAT00159 | 36 | 9324 | 6760 | 14 | [MARTX1] hypothetical protein | * |
| BAT00160 | 24 | 5361 | 0 | 4.5 | [MARTX1] hypothetical protein | * |
| BAT00161 | 2 | 1710 | 0 | 1.2 | Hemolysin activator protein, hlyb family | * |
| BAT00289-290 | 4 | 1536 | 3 | 2.6 | Rtxa | * |
| BAT00290-end | 199 | 3672 | 0 | 54.2 | Rtx | * |
| BAT00370 | 2 | 1158 | 0 | 1.7 | Hemolysin-type calcium-binding protein | * |
| BAT00371 | 11 | 1341 | 0 | 8.2 | Protein containing Type 1 secretion,  C-terminal domain (RTX) | |
| BAT00557 | 100 | 1071 | 0 | 93.4 | Insecticidal toxin protein |  |
| BAT00890-891 | 39 | 1371 | 49 | 29.5 | Rhs | * |
| BAT00891 | 5 | 123 | 0 | 40.7 | Probable RHS | * |
| BAT00892-894 | 93 | 4059 | 365 | 25.2 | Rhs | * |
| BAT01108 | 3 | 2274 | 0 | 1.3 | Cell well associated rhsd protein precursor | * |
| BAT01109 | 17 | 8529 | 0 | 2 | Toxin complex/plasmid virulence protein | * |
| BAT01110 | 10 | 5343 | 0 | 1.9 | Insecticidal toxin complex protein | * |
| BAT01111 | 2 | 2463 | 2177 | 7 | Probable RHS | * |
| BAT01112 | 1 | 135 | 0 | 7.4 | Probable RHS |  |
| BAT01113 | 1 | 3273 | 3020 | 4 | Probable RHS | * |
| BAT01114 | 2 | 1596 | 0 | 1.3 | Insecticidal toxin complex protein | * |
| BAT01115 | 0 | 726 | 76 | 0 | YD repeat-containing protein | * |
| BAT01116 | 0 | 759 | 0 | 0 | Hypothetical protein (RHS) | * |
| BAT01117 | 0 | 1530 | 0 | 0 | YD repeat-containing protein | * |
| BAT01118 | 0 | 1443 | 0 | 0 | YD repeat-containing protein | * |
| BAT01120 | 0 | 1542 | 0 | 0 | YD repeat-containing protein | * |
| BAT01122 | 0 | 1158 | 359 | 0 | Insecticidal toxin complex protein | * |
| BAT01123 | 0 | 591 | 0 | 0 | Probable RHS | * |
| BAT01124 | 0 | 1533 | 0 | 0 | YD repeat-containing protein | * |
| BAT01125 | 0 | 852 | 659 | 0 | Probable RHS | * |
| BAT01126 | 0 | 756 | 0 | 0 | Probable RHS | * |
| BAT01127 | 0 | 105 | 0 | 0 | Probable RHS |  |
| BAT01128 | 4 | 204 | 0 | 19.6 | Probable RHS |  |
| BAT01129 | 1 | 210 | 0 | 4.8 | Probable RHS |  |
| BAT01130 | 0 | 186 | 0 | 0 | Probable RHS |  |
| BAT01309 | 0 | 495 | 0 | 0 | Probable RHS | * |
| BAT01311 | 0 | 627 | 0 | 0 | Probable RHS | * |
| BAT01314 | 0 | 801 | 0 | 0 | Probable RHS | * |
| BAT01451 | 43 | 2844 | 1594 | 34.4 | [MARTX2] RTX toxin RtxA | * |
| BAT01452 | 39 | 3396 | 0 | 11.5 | [MARTX2] Hypothetical protein | * |
| BAT01453 | 8 | 2862 | 1899 | 8.3 | [MARTX2] Hypothetical protein |  |
| BAT01454 | 39 | 11352 | 7602 | 10.4 | [MARTX2] Peptidase C80, RTX self-cleaving toxin | * |
| BAT01455 | 51 | 4704 | 0 | 10.8 | [MARTX2] Peptidase C80, RTX self-cleaving toxin | * |
| BAT01456 | 23 | 9663 | 3136 | 3.5 | [MARTX2] Peptidase C80, RTX self-cleaving toxin | * |
| BAT01457 | 7 | 2286 | 646 | 4.3 | [MARTX2] RTX toxin rtxa-like protein | * |
| BAT01458 | 5 | 363 | 0 | 13.8 | [MARTX2] hypothetical protein | * |
| BAT01459 | 9 | 3069 | 2035 | 8.7 | [MARTX2] RTX toxin rtxa |  |
| BAT01461 | 0 | 129 | 0 | 0 | [MARTX2] Hypothetical protein |  |
| BAT01485 | 50 | 1515 | 0 | 33 | YD repeat-containing protein | * |
| BAT01505 | 3 | 6690 | 4118 | 1.2 | Rhs family protein | * |
| BAT01658 | 29 | 4743 | 0 | 6.1 | Hemolysin-type calcium-binding region | * |
| BAT01946 | 37 | 1488 | 0 | 24.9 | YD repeat-containing protein | * |
| BAT01950 | 2 | 819 | 0 | 2.4 | Probable RHS | * |
| BAT01957 | 0 | 831 | 0 | 0 | Probable RHS | * |
| BAT01959 | 0 | 1284 | 0 | 0 | Tccc3 | * |
| BAT01961 | 0 | 1140 | 0 | 0 | Tccc4 |  |
| BAT01964 | 19 | 1206 | 0 | 15.8 | Tccc4 | * |
| BAT02031 | 22 | 1032 | 0 | 21.3 | Insecticidal toxin complex protein |  |
| BAT02033 | 117 | 1044 | 0 | 112.1 | Insecticidal toxin complex-like protein | * |
| BAT02222 | 3 | 813 | 0 | 3.7 | Probable RHS | * |
| BAT02223 | 4 | 4041 | 3881 | 25 | Probable RHS | * |
| BAT02224 | 18 | 585 | 0 | 30.8 | Probable RHS |  |
| BAT02225 | 13 | 5763 | 3898 | 7 | Insecticidal toxin complex protein | * |
| BAT02228 | 0 | 3273 | 2056 | 0 | Insecticidal toxin complex protein | * |
| BAT02229 | 0 | 1215 | 0 | 0 | Insecticidal toxin complex protein | * |
| BAT02230 | 0 | 1545 | 0 | 0 | YD repeat-containing protein | * |
| BAT02231 | 0 | 3264 | 2097 | 0 | Insecticidal toxin complex protein | * |
| BAT02232 | 0 | 1197 | 0 | 0 | Insecticidal toxin complex protein TccC3 | * |
| BAT02233 | 0 | 1521 | 0 | 0 | YD repeat-containing protein | * |
| BAT02234 | 0 | 1548 | 0 | 0 | Insecticidal toxin complex protein TccC (Toxin complex protein) | * |
| BAT02235 | 0 | 240 | 20 | 0 | Probable RHS | * |
| BAT02236 | 0 | 351 | 0 | 0 | Probable RHS |  |
| BAT02238 | 0 | 825 | 0 | 0 | Probable RHS | * |
| BAT02239 | 0 | 1578 | 1462 | 0 | Probable RHS | * |
| BAT02240 | 0 | 3000 | 2266 | 0 | Probable RHS | * |
| BAT02242 | 0 | 492 | 0 | 0 | Insecticidal toxin complex protein | * |
| BAT02243 | 0 | 354 | 265 | 0 | Probable RHS |  |
| BAT02244 | 2 | 810 | 0 | 2.5 | Probable RHS |  |
| Gcontig00696_0 | 0 | 1023 | 0 | 0 | YD repeat-containing protein | * |
| Gcontig00723_0 | 9 | 702 | 0 | 12.8 | Sugar-binding protein (RHS) | * |
| Gcontig00727_0 | 27 | 660 | 0 | 40.9 | Insecticidal toxin complex protein | * |
| Gcontig00791_2 | 3 | 465 | 0 | 6.5 | YD repeat-containing protein partial |  |
| Gcontig00849_0 | 10 | 351 | 0 | 28.5 | Outer membrane channel lipoprotein (RTX) |  |
| Gorf51_glimmer3 | 13 | 1548 | 0 | 8.4 | YD repeat-containing protein |  |
| Gorf52_glimmer3 | 6 | 1548 | 766 | 7.7 | YD repeat-containing protein |  |
| scaffold00001_63 | 3 | 2535 | 1494 | 2.9 | Hypothetical protein [MARTX] |  |
| scaffold00001_64 | 2 | 1485 | 0 | 1.3 | Conserved hypothetical protein [MARTX] | * |
| scaffold00001_66 | 37 | 5430 | 20 | 6.8 | Filamentous hemagglutinin family, N-terminal domain-containing protein [MARTX] | * |
| scaffold00001_67 | 1 | 744 | 20 | 1.4 | Hypothetical protein [MARTX] | * |
| scaffold00001_68 | 29 | 2796 | 177 | 11.1 | Peptidase C80 family [MARTX] | * |
| scaffold00001_69 | 24 | 3690 | 335 | 7.2 | Peptidase C80 family [MARTX] | * |
| scaffold00001_70 | 22 | 6405 | 31 | 3.5 | Peptidase C80 family [MARTX] | * |
| scaffold00007_28 | 14 | 2349 | 54 | 6.1 | Peptidase C80 family [MARTX] | * |
| scaffold00007_30 | 14 | 3105 | 51 | 4.6 | Peptidase C80 family [MARTX] | * |
| scaffold00033_0 | 0 | 543 | 231 | 0 | YD repeat-containing protein |  |
| scaffold00033_1 | 2 | 3645 | 0 | 0.5 | YD repeat-containing protein | * |
| scaffold00033_2 | 5 | 843 | 0 | 5.9 | Insecticidal toxin complex protein |  |
| scaffold00033_3 | 0 | 891 | 0 | 0 | Plasmid 28.1 kda A protein |  |
| scaffold00033_4 | 1 | 1953 | 20 | 0.5 | Insecticidal toxin complex protein | * |
| scaffold00033_6 | 2 | 276 | 0 | 7.2 | Virulence plasmid 28.1 kda A protein |  |
| scaffold00033_7 | 2 | 834 | 0 | 2.4 | Virulence plasmid 28.1 kda A protein | * |
| scaffold00036_14 | 0 | 1680 | 0 | 0 | Hemolysin-type calcium-binding conserved site | * |
| scaffold00080_9 | 7 | 2841 | 725 | 3.3 | Hypothetical protein [MARTX] |  |
| scaffold00083_0 | 35 | 4236 | 401 | 9.1 | YD repeat-containing protein partial | * |
| scaffold00108_0 | 29 | 1932 | 1032 | 32.2 | YD repeat-containing protein | * |
| Acontig00027_1 | 8 | 5322 | 0 | 1.5 | YD repeat-containing protein | * |
| Acontig00027_2 | 0 | 1659 | 0 | 0 | Insecticidal toxin complex protein |  |
| Acontig00030_0 | 4 | 4914 | 0 | 0.8 | Plasmid 28.1 kda A protein | * |
| Acontig03871_4 | 0 | 1023 | 0 | 0 | RHS repeat-associated core domain protein containing protein | * |
| Acontig03872_2 | 8 | 1053 | 0 | 7.6 | YD repeat-containing protein | * |
| Acontig104979_0 | 0 | 1371 | 0 | 0 | Virulence plasmid 28.1 kda A protein | * |
| Acontig134087_0 | 0 | 183 | 0 | 0 | YD repeat-containing protein |  |
| Acontig134087_1 | 3 | 1074 | 0 | 2.8 | YD repeat-containing protein partial | * |
| Acontig206100_1 | 12 | 414 | 0 | 29 | Rhsb protein |  |
| Acontig211660_0 | 26 | 594 | 0 | 43.8 | YD repeat-containing protein |  |
| Acontig21192_0 | 34 | 2445 | 0 | 13.9 | Peptidase C80 (MARTX) | * |
| Acontig32555_0 | 3 | 1686 | 0 | 1.8 | Hemolysin secretion/activation protein shlb family | * |
| Acontig47013_1 | 11 | 1545 | 0 | 7.1 | YD repeat-containing protein | * |
| Acontig54005_0 | 3 | 1554 | 0 | 1.9 | RHS repeat-associated core domain protein-containing protein | |
| Acontig64332_0 | 0 | 120 | 0 | 0 | Conserved hypothetical protein (RHS) |  |
| Acontig64332_1 | 4 | 1551 | 0 | 2.6 | RHS repeat-associated core domain protein containing protein | * |
| Acontig71420_1 | 6 | 1257 | 0 | 4.8 | YD repeat-containing protein | * |
| Acontig80766_0 | 4 | 1239 | 0 | 3.2 | RHS repeat-associated core domain protein containing protein | * |
| Acontig88396_0 | 1 | 765 | 0 | 1.3 | YD repeat-containing protein | * |
| Acontig96355_1 | 0 | 129 | 0 | 0 | Hypothetical protein (RHS) |  |

*Genes with a different codon usage, most likely acquired through horizontal gene transfer
