# Supplementary material for: Abundant toxin-related genes in the genomes of beneficial symbionts from deep-sea hydrothermal vent mussels
Source: eLife. 2015 Sep 15;4:e07966. doi: 10.7554/eLife.07966 (PMC4612132; doi:10.7554/eLife.07966)
Supplement: Supplementary file 1. — (A) Number of mobile elements in the genomes compared in this study. (B) Genomes with toxin-related genes (TRGs) similar to those of the sulfur-oxidizing (SOX) symbionts of Bathymodiolus. The number of genes per TRGs class is shown. (C) p-values obtained with one-way Permanova were corrected with Bonferroni correction for multiple testing. Number of TRGs per genome was normalized to the total gene count. (D) Transcriptome counts of three individuals from B. azoricus and three individuals from B. sp. were mapped to their respective reference genomes with Rockhopper. Expression values of TRGs were normalized to the expression of RubisCO. (E) Samples used in this study. (F) Primer sequences and annealing temperatures used to detect genome rearrangements. (G) Metagenomes and metatranscriptomes enriched in SUP05 from oxygen minimum zones (OMZ) or hydrothermal vents. (H) Amino acid sequences from the following genomes were used in the reference database for proteomic analysis (IncDB). The genomes belong to relatives of the SOX and methane-oxidizing (MOX) symbionts of B. azoricus, as well as the mussel host. (I) Details of expressed toxin-related proteins identified with proteomics. The values are given in % normalized spectral abundance factor (NSAF), which is a normalized spectral abundance factor that gives the relative abundance of a protein in a sample in %. DOI: http://dx.doi.org/10.7554/eLife.07966.023 [file elife07966s002.docx]

# Supplementary File 1

Supplementary File 1A. Number of mobile elements in the genomes compared in this study

| **Genome** | **# Transposases** | **# Integrases** | **# Restriction-modification systems** |
| --- | --- | --- | --- |
| **BspSym** | 17 | 5 | 18 |
| **BazSymA** | 23 | 4 | 22 |
| **BazSymB** | 13 | 3 | 10 |
| **SUP05^*^** | 14 | 1 | 1 |
| ***Candidatus* Ruthia magnifica^#^** | 0 | 0 | 0 |
| ***Candidatus* Vesicomyosocius okutanii^+^** | 0 | 0 | 0 |

^*^(Walsh et al., 2009)

**^#^**(Newton et al., 2007)

^+^(Kuwahara et al., 2007)

Supplementary Table1B. Genomes with TRGs similar to those of the SOX symbionts of *Bathymodiolus.* The number of genes per TRGs class is shown.

| **Genome** | **Class** | **YD^#^** | **RTX^#^** | **MARTX^#^** | **NCBI Taxon ID** | **Gene count** | **Lifestyle**  **-** | | |
| --- | --- | --- | --- | --- | --- | --- | --- | --- | --- |
| *Acinetobacter baumannii* ATCC 17978 | Gammaproteobacteria | 0 | 2 | 0 | 400667 | 3464 | Ext | P | * |
| *Agrobacterium vitis* S4 | Alphaproteobacteria | 0 | 2 | 0 | 311402 | 5455 | Ext | P | * |
| *Anabaena cylindrica* PCC 7122 | Cyanophyceae | 0 | 8 | 0 | 272123 | 6258 | FL | NP | * |
| *Anabaena variabilis* ATCC 29413 | Cyanophyceae | 0 | 6 | 0 | 240292 | 5772 | FL | NP | * |
| *Azospirillum brasilense* Sp245 | Alphaproteobacteria | 0 | 13 | 0 | 192 | 7962 | Ext | NP | * |
| *Azospirillum lipoferum* 4B | Alphaproteobacteria | 0 | 4 | 0 | 862719 | 6349 | Ext | NP | * |
| *Bordetella bronchiseptica* RB50 | Betaproteobacteria | 0 | 1 | 2 | 257310 | 5086 | Ext | P | * |
| *Bordetella parapertussis* 12822 | Betaproteobacteria | 0 | 1 | 1 | 257311 | 4447 | Ext | P | * |
| *Bordetella pertussis* CS | Betaproteobacteria | 0 | 1 | 1 | 1017264 | 3516 | Ext | P | * |
| *Bradyrhizobium japonicum* USDA 110 | Alphaproteobacteria | 0 | 3 | 0 | 224911 | 8402 | Int | NP | * |
| Caldicellulosiruptor obsidiansis OB47 | Clostridia | 0 | 1 | 0 | 608506 | 2389 | FL | NP | * |
| Candidatus Hamiltonella defensa 5AT (Acyrthosiphon pisum) | Gammaproteobacteria | 0 | 2 | 0 | 572265 | 2200 | Int | P |  |
| Candidatus Nitrospira defluvii | Nitrospira | 0 | 1 | 0 | 330214 | 4317 | FL | NP |  |
| Chlorobium limicola DSM 245 | Chlorobia | 0 | 5 | 0 | 290315 | 2576 | FL | NP |  |
| Chlorobium phaeobacteroides BS1 | Chlorobia | 0 | 1 | 0 | 331678 | 2611 | FL | NP |  |
| Collimonas fungivorans Ter331 | Betaproteobacteria | 0 | 2 | 2 | 1005048 | 4493 | Ext | NP |  |
| Dechlorosoma suillum PS | Betaproteobacteria | 0 | 4 | 0 | 640081 | 3539 | FL | NP |  |
| Desulfobacula toluolica Tol2 | Deltaproteobacteria | 0 | 3 | 0 | 651182 | 4435 | FL | NP |  |
| Desulfovibrio alaskensis G20 | Deltaproteobacteria | 0 | 1 | 0 | 207559 | 3874 | FL | NP |  |
| *Filifactor alocis* ATCC 35896 | Clostridia | 0 | 1 | 0 | 546269 | 1709 | Ext | NP | * |
| *Francisella tularensis* subsp. tularensis TIGB03 | Gammaproteobacteria | 0 | 1 | 0 | 1001542 | 1850 | Int | P |  |
| *Gallibacterium anatis* UMN179 | Gammaproteobacteria | 0 | 3 | 3 | 1005058 | 2576 | Ext | P |  |
| *Herbaspirillum seropedicae* SmR1 | Betaproteobacteria | 0 | 3 | 2 | 757424 | 4799 | Int | NP | * |
| *Methylotenera mobilis* JLW8 | Betaproteobacteria | 0 | 1 | 0 | 583345 | 2400 | FL | NP | * |
| *Neisseria meningitidis* 053442 | Betaproteobacteria | 0 | 2 | 0 | 374833 | 2116 | Ext | P | * |
| *Neisseria meningitidis* MC58 | Betaproteobacteria | 0 | 3 | 4 | 122586 | 2226 | Ext | P | * |
| *Nostoc punctiforme* PCC 73102 | unclassified | 0 | 8 | 0 | 63737 | 6791 | Ext | NP | * |
| *Oscillatoria acuminata* PCC 6304 | unclassified | 0 | 15 | 0 | 56110 | 6101 | FL | NP | * |
| *Paracoccus denitrificans* PD1222 | Alphaproteobacteria | 0 | 8 | 0 | 318586 | 5158 | FL | NP | * |
| *Phaeobacter gallaeciensis* DSM 17395 = CIP 105210 | Alphaproteobacteria | 0 | 5 | 0 | 391619 | 3960 | FL | NP | * |
| *Phenylobacterium zucineum* HLK1 | Alphaproteobacteria | 0 | 6 | 0 | 450851 | 3899 | Int | P |  |
| *Pseudomonas aeruginosa* PAO1 | Gammaproteobacteria | 0 | 0 | 2 | 208964 | 5671 | Ext | P | * |
| *Pseudomonas putida* S16 | Gammaproteobacteria | 0 | 2 | 0 | 1042876 | 5307 | FL | NP | * |
| *Ralstonia solanacearum* Po82 | Betaproteobacteria | 0 | 4 | 4 | 1031711 | 5080 | Ext | P | * |
| *Rhizobium etli* CIAT 652 | Alphaproteobacteria | 0 | 3 | 0 | 491916 | 6116 | Int | NP | * |
| *Rhodopseudomonas palustris* HaA2 | Alphaproteobacteria | 0 | 4 | 0 | 316058 | 4788 | FL | NP | * |
| *Roseobacter denitrificans* OCh 114 | Alphaproteobacteria | 0 | 6 | 0 | 375451 | 4201 | Ext | NP | * |
| *Roseobacter litoralis* Och 149 | Alphaproteobacteria | 0 | 7 | 0 | 391595 | 4668 | Ext | NP | * |
| *Shewanella violacea* DSS12 | Gammaproteobacteria | 0 | 1 | 0 | 637905 | 4515 | FL | NP |  |
| *Sphingobium japonicum* UT26S | Alphaproteobacteria | 0 | 3 | 0 | 452662 | 4460 | FL | NP |  |
| *Streptococcus sanguinis* SK36 | Bacilli | 0 | 1 | 0 | 388919 | 2367 | Ext | P | * |
| *Vibrio vulnificus* YJ016 | Gammaproteobacteria | 0 | 1 | 0 | 196600 | 5202 | Ext | P | * |
| *Xylella fastidiosa* Temecula1 | Gammaproteobacteria | 0 | 4 | 0 | 183190 | 2102 | Ext | P | * |
| *Rhodococcus jostii* RHA1 | Actinobacteria | 1 | 0 | 0 | 101510 | 9242 | FL | NP |  |
| *Acaryochloris marina* MBIC11017 | unclassified | 1 | 14 | 0 | 329726 | 8488 | Ext | NP | * |
| *Herpetosiphon aurantiacus* DSM 785 | Chloroflexi | 1 | 0 | 0 | 316274 | 5654 | FL | NP |  |
| *Trichodesmium erythraeum* IMS101 | unclassified | 1 | 14 | 0 | 203124 | 5156 | FL | NP | * |
| *Alcanivorax dieselolei* B5 | Gammaproteobacteria | 1 | 0 | 0 | 930169 | 4470 | FL | NP | * |
| *Pelobacter propionicus* DSM 2379 | Deltaproteobacteria | 1 | 1 | 0 | 338966 | 3949 | FL | NP | * |
| *Geobacter sulfurreducens* PCA | Deltaproteobacteria | 1 | 1 | 0 | 243231 | 3552 | FL | NP | * |
| *Ralstonia eutropha* H16 | Betaproteobacteria | 2 | 2 | 0 | 381666 | 6718 | FL | NP |  |
| *Methanoregula formicicum* SMSP | Methanomicrobia | 1 | 0 | 0 | 593750 | 2924 | FL | NP | * |
| *Nitrosospira multiformis* ATCC 25196 | Betaproteobacteria | 1 | 0 | 0 | 323848 | 2885 | FL | NP | * |
| *Caldicellulosiruptor saccharolyticus* DSM 8903 | Clostridia | 1 | 0 | 0 | 351627 | 2834 | FL | NP |  |
| *Caldicellulosiruptor kronotskyensis* 2002 | Clostridia | 1 | 0 | 0 | 632348 | 2642 | FL | NP |  |
| *Rahnella aquatilis* HX2 | Gammaproteobacteria | 3 | 0 | 2 | 1151116 | 5060 | Ext | NP |  |
| *Listeria monocytogenes* HCC23 | Bacilli | 2 | 0 | 0 | 552536 | 3059 | Int | P |  |
| *Geobacter uraniireducens* Rf4 | Deltaproteobacteria | 3 | 1 | 0 | 351605 | 4542 | FL | NP | * |
| *Erwinia pyrifoliae* DSM 12163 | Gammaproteobacteria | 3 | 0 | 2 | 644651 | 4134 | Ext | NP | * |
| *Shewanella piezotolerans* WP3 | Gammaproteobacteria | 4 | 2 | 0 | 225849 | 5047 | FL | NP |  |
| *Shewanella baltica* OS223 | Gammaproteobacteria | 4 | 0 | 0 | 407976 | 4622 | FL | NP | * |
| *Pseudomonas entomophila* L48 | Gammaproteobacteria | 5 | 1 | 1 | 384676 | 5293 | Ext | P | * |
| *Methylomonas methanica* MC09 | Gammaproteobacteria | 5 | 0 | 0 | 857087 | 4664 | FL | NP | * |
| *Hahella chejuensis* KCTC 2396 | Gammaproteobacteria | 8 | 6 | 0 | 349521 | 6875 | FL | NP |  |
| *Burkholderia pseudomallei* 1710b | Betaproteobacteria | 8 | 0 | 2 | 320372 | 6436 | Int | P | * |
| *Burkholderia rhizoxinica* HKI 454 | Betaproteobacteria | 5 | 0 | 2 | 882378 | 3938 | Int | NP | * |
| *Pseudomonas fluorescens* A506 | Gammaproteobacteria | 7 | 1 | 2 | 1037911 | 5426 | Ext | NP | * |
| Yersinia pestis Antiqua | Gammaproteobacteria | 6 | 0 | 1 | 360102 | 4576 | Int | P | * |
| Candidatus *Amoebophilus asiaticus* 5a2 | unclassified | 2 | 0 | 0 | 452471 | 1487 | Int | P |  |
| *Pseudomonas fluorescens* F113 | Gammaproteobacteria | 10 | 1 | 3 | 1114970 | 5952 | Ext | NP | * |
| *Shewanella denitrificans* OS217 | Gammaproteobacteria | 7 | 0 | 0 | 318161 | 3935 | FL | NP |  |
| *Yersinia pseudotuberculosis* IP 31758 | Gammaproteobacteria | 9 | 0 | 2 | 349747 | 4529 | Int | P | * |
| *Cellvibrio japonicus* Ueda107 | Gammaproteobacteria | 9 | 1 | 0 | 498211 | 3811 | FL | NP |  |
| *Xenorhabdus nematophila* ATCC 19061 | Gammaproteobacteria | 12 | 2 | 1 | 406817 | 4583 | Int | P | * |
| *Photorhabdus luminescens* subsp. laumondii TTO1 | Gammaproteobacteria | 17 | 7 | 4 | 243265 | 5052 | Ext | P |  |
| BazSymB | Gammaproteobacteria | 14 | 2 | 10 | NA | 1802 | Int | NP |  |
| BazSymA | Gammaproteobacteria | 16 | 0 | 1 | NA | 2007 | Int | NP |  |
| BspSym | Gammaproteobacteria | 33 | 8 | 19 | 174145 | 2225 | Int | NP |  |

FL = free-living, Ext = extracellular host associated, Int = intracellular host associated, P = pathogen, NP = non-pathogen.

^#^The sum of the genes that had a significant BLAST hit against the YD, RTX and MARTX genes of the SOX symbionts.

*Found in biofilms.

**Supplementary File 1C. P-values obtained with one-way Permanova were corrected with Bonferroni correction for multiple testing.** **Number of TRGs per genome was normalized to the total gene count**

|  | **Class** | **Order** | **Family** |
| --- | --- | --- | --- |
| **YD** | 0.3154 | 0.2731 | 0.0513 |
| **RTX** | 0.0812 | 0.0159* | 0.214 |
| **MARTX** | 0.5479 | 0.3415 | 0.3093 |

**Supplementary File 1D. Transcriptome counts of three individuals from *B. azoricus* and three individuals from *B*. sp. were mapped to their respective reference genomes with Rockhopper. Expression values of TRGs were normalized to the expression of RubisCO.**

| **Genome** | **Identifier** | **Annotation** | **Class** | **Rep* 1** | **Rep* 2** | **Rep* 3** |
| --- | --- | --- | --- | --- | --- | --- |
| BazSymA | Acontig00027_1 | YD repeat-containing protein | RHS | 0.15 | 0.39 | 0.04 |
| BazSymA | Acontig00030_0 | Plasmid 28.1 kda A protein | RHS | 0.07 | 0.11 | 0.03 |
| BazSymA | Acontig03871_4 | RHS repeat-associated core domain protein containing protein | RHS | 0.19 | 0.71 | 0.05 |
| BazSymA | Acontig03872_2 | YD repeat-containing protein | RHS | 0.47 | 1.69 | 0.19 |
| BazSymA | Acontig104979_0 | Virulence plasmid 28.1 kda A protein | RHS | 0.00 | 0.03 | 0.00 |
| BazSymA | Acontig134087_1 | YD repeat-containing protein | RHS | 0.14 | 0.74 | 0.05 |
| BazSymA | Acontig21192_0 | Peptidase C80 family [MARTX] | MARTX | 0.61 | 1.81 | 0.17 |
| BazSymA | Acontig47013_1 | YD repeat-containing protein | RHS | 0.53 | 1.83 | 0.14 |
| BazSymA | Acontig64332_1 | RHS repeat-associated core domain protein containing protein | RHS | 0.32 | 1.27 | 0.08 |
| BazSymA | Acontig71420_1 | YD repeat-containing protein | RHS | 0.28 | 0.97 | 0.07 |
| BazSymA | Acontig80766_0 | RHS repeat-associated core domain protein containing protein | RHS | 0.25 | 0.87 | 0.05 |
| BazSymA | Acontig88396_0 | YD repeat-containing protein | RHS | 0.41 | 1.75 | 0.07 |
| BazSymA | Acontig211660_0 | YD repeat-containing protein | RHS | 0.44 | 1.55 | 0.15 |
| BazSymA | Acontig134087_0 | YD repeat-containing protein | RHS | 0.35 | 1.68 | 0.10 |
| BazSymA | Acontig54005_0 | RHS repeat-associated core domain protein-containing protein | RHS | 0.18 | 0.82 | 0.06 |
| BazSymA | Acontig64332_0 | Probable RHS | probable RHS | 0.41 | 1.84 | 0.18 |
| BazSymA | Acontig206100_1 | RhsB protein | RHS | 0.10 | 0.31 | 0.04 |
| BazSymA | Acontig00027_2 | Insecticidal toxin complex protein | RHS | 0.07 | 0.14 | 0.04 |
| BazSymA | Acontig96355_1 | Probable RHS | probable RHS | 0.24 | 1.10 | 0.04 |
| BazSymA | Acontig32555_0 | Hemolysin secretion/activation protein shlb family | RTX activator | 0.11 | 0.50 | 0.04 |
| BazSymB | Gcontig00696_0 | YD repeat-containing protein | RHS | 0.43 | 1.46 | 0.09 |
| BazSymB | Gcontig00723_0 | Probable RHS | probable RHS | 1.20 | 3.78 | 0.26 |
| BazSymB | Gcontig00727_0 | Insecticidal toxin protein | RHS | 1.37 | 4.39 | 0.25 |
| BazSymB | scaffold00001_64 | Conserved hypothetical protein [MARTX] | MARTX | 0.57 | 1.55 | 0.15 |
| BazSymB | scaffold00001_66 | Filamentous hemagglutinin family N-terminal domain-containing protein | MARTX | 1.03 | 2.80 | 0.24 |
| BazSymB | scaffold00001_67 | Hypothetical protein [MARTX] | MARTX | 0.90 | 2.83 | 0.23 |
| BazSymB | scaffold00001_68 | Peptidase C80 family [MARTX] | MARTX | 1.00 | 3.01 | 0.22 |
| BazSymB | scaffold00001_69 | Peptidase C80 family [MARTX] | MARTX | 1.20 | 3.62 | 0.37 |
| BazSymB | scaffold00001_70 | Peptidase C80 family [MARTX] | MARTX | 0.84 | 2.12 | 0.22 |
| BazSymB | scaffold00007_28 | Peptidase C80 | MARTX | 1.15 | 3.07 | 0.26 |
| BazSymB | scaffold00007_30 | Peptidase C80 | MARTX | 0.56 | 1.85 | 0.17 |
| BazSymB | scaffold00033_1 | YD repeat-containing protein | RHS | 0.25 | 0.57 | 0.06 |
| BazSymB | scaffold00033_4 | Insecticidal toxin complex protein | RHS | 0.27 | 0.33 | 0.14 |
| BazSymB | scaffold00033_7 | Virulence plasmid 28.1 kda A protein | RHS | 0.19 | 0.29 | 0.06 |
| BazSymB | scaffold00036_14 | Hemolysin-type calcium-binding conserved site | RTX | 0.23 | 0.70 | 0.04 |
| BazSymB | scaffold00083_0 | YD repeat-containing protein | RHS | 1.33 | 2.34 | 0.15 |
| BazSymB | scaffold00108_0 | YD repeat-containing protein | RHS | 0.64 | 2.04 | 0.15 |
| BazSymB | scaffold00080_9 | Peptidase C80 | MARTX | 0.17 | 0.43 | 0.05 |
| BazSymB | scaffold00001_63 | Hypothetical protein [MARTX] | MARTX | 0.18 | 0.43 | 0.04 |
| BazSymB | Gcontig00791_2 | YD repeat-containing protein | RHS | 0.77 | 2.78 | 0.25 |
| BazSymB | Gorf51_glimmer3 | YD repeat-containing protein | RHS | 0.96 | 3.30 | 0.23 |
| BazSymB | scaffold00033_6 | Virulence plasmid 28.1 kda A protein | RHS | 0.39 | 0.77 | 0.15 |
| BazSymB | Gorf52_glimmer3 | YD repeat-containing protein | RHS | 0.48 | 1.84 | 0.15 |
| BazSymB | scaffold00033_2 | Insecticidal toxin complex protein | RHS | 0.47 | 1.00 | 0.09 |
| BazSymB | scaffold00033_3 | Plasmid 28.1 kda A protein | RHS | 0.16 | 0.26 | 0.06 |
| BazSymB | scaffold00033_0 | YD repeat-containing protein | RHS | 0.37 | 1.01 | 0.05 |
| BazSymB | Gcontig00849_0 | Hemolysin type | RTX | 1.25 | 4.18 | 0.42 |
| BspSym | BAT00155 | Hypothetical protein [MARTX] | MARTX | 0.73 | 0.01 | 0.00 |
| BspSym | BAT00157 | Hypothetical protein [MARTX] | MARTX | 0.58 | 0.01 | 0.00 |
| BspSym | BAT00370 | Hemolysin-type calcium-binding protein | RTX | 0.50 | 0.02 | 0.02 |
| BspSym | BAT00890-891 | Rhs | RHS | 0.78 | 0.06 | 0.04 |
| BspSym | BAT00891 | Probable RHS | Probable RHS | 0.83 | 0.03 | 0.00 |
| BspSym | BAT00892-894 | Rhs | RHS | 0.35 | 0.02 | 0.01 |
| BspSym | BAT01108 | Cell well associated rhsd protein precursor | RHS | 0.47 | 0.09 | 0.11 |
| BspSym | BAT01109 | Toxin complex/plasmid virulence protein | RHS | 0.51 | 0.07 | 0.13 |
| BspSym | BAT01110 | Insecticidal toxin complex protein | RHS | 0.47 | 0.04 | 0.05 |
| BspSym | BAT01111 | Probable RHS | probable RHS | 0.34 | 0.01 | 0.00 |
| BspSym | BAT01113 | Probable RHS | probable RHS | 0.25 | 0.00 | 0.00 |
| BspSym | BAT01114 | Insecticidal toxin protein | RHS | 0.41 | 0.00 | 0.00 |
| BspSym | BAT01115 | YD repeat-containing protein | RHS | 0.65 | 0.01 | 0.00 |
| BspSym | BAT01116 | Hypothetical protein [RHS] | RHS | 0.41 | 0.00 | 0.00 |
| BspSym | BAT01117 | YD repeat-containing protein | RHS | 0.63 | 0.00 | 0.00 |
| BspSym | BAT01118 | YD repeat-containing protein | RHS | 0.45 | 0.00 | 0.00 |
| BspSym | BAT01120 | YD repeat-containing protein | RHS | 0.59 | 0.00 | 0.00 |
| BspSym | BAT01122 | Insecticidal toxin complex protein | RHS | 0.35 | 0.00 | 0.00 |
| BspSym | BAT01123 | Probable RHS | probable RHS | 0.62 | 0.00 | 0.00 |
| BspSym | BAT01124 | YD repeat-containing protein | RHS | 0.59 | 0.01 | 0.01 |
| BspSym | BAT01125 | Probable RHS | probable RHS | 1.48 | 0.01 | 0.00 |
| BspSym | BAT01126 | Probable RHS | probable RHS | 0.56 | 0.01 | 0.00 |
| BspSym | BAT01309 | Probable RHS | probable RHS | 0.44 | 0.01 | 0.00 |
| BspSym | BAT01311 | Probable RHS | probable RHS | 0.35 | 0.01 | 0.01 |
| BspSym | BAT01314 | Probable RHS | probable RHS | 0.38 | 0.01 | 0.00 |
| BspSym | BAT01451 | RTX toxin RtxA | MARTX | 0.59 | 0.02 | 0.02 |
| BspSym | BAT01452 | Hypothetical protein [MARTX] | MARTX | 0.49 | 0.01 | 0.00 |
| BspSym | BAT01454 | Peptidase C80 | MARTX | 0.17 | 0.01 | 0.01 |
| BspSym | BAT01455 | Peptidase C80 | MARTX | 0.28 | 0.01 | 0.01 |
| BspSym | BAT01456 | Peptidase C80 | MARTX | 0.32 | 0.01 | 0.01 |
| BspSym | BAT01457 | RTX toxin rtxa-like protein | MARTX | 0.52 | 0.02 | 0.01 |
| BspSym | BAT01458 | Hypothetical protein | MARTX | 1.29 | 0.07 | 0.01 |
| BspSym | BAT01485 | YD repeat-containing protein | RHS | 0.35 | 0.00 | 0.00 |
| BspSym | BAT01505 | Rhs family protein | RHS | 0.12 | 0.00 | 0.00 |
| BspSym | BAT01658 | Hemolysin-type calcium-binding region | RTX | 0.69 | 0.04 | 0.05 |
| BspSym | BAT01946 | YD repeat-containing protein | RHS | 0.47 | 0.01 | 0.00 |
| BspSym | BAT01950 | Probable RHS | probable RHS | 0.36 | 0.00 | 0.01 |
| BspSym | BAT01957 | Probable RHS | probable RHS | 0.35 | 0.00 | 0.00 |
| BspSym | BAT01959 | Tccc3 | RHS | 0.43 | 0.01 | 0.01 |
| BspSym | BAT01964 | Tccc4 | RHS | 0.46 | 0.00 | 0.00 |
| BspSym | BAT02033 | Insecticidal toxin complex-like protein | RHS | 0.70 | 0.00 | 0.00 |
| BspSym | BAT02222 | Probable RHS | probable RHS | 0.27 | 0.00 | 0.00 |
| BspSym | BAT02223 | Probable RHS | probable RHS | 0.03 | 0.00 | 0.00 |
| BspSym | BAT02225 | Insecticidal toxin protein | RHS | 0.20 | 0.00 | 0.00 |
| BspSym | BAT02228 | Insecticidal toxin protein | RHS | 0.43 | 0.01 | 0.00 |
| BspSym | BAT02229 | Insecticidal toxin protein | RHS | 0.28 | 0.00 | 0.00 |
| BspSym | BAT02230 | YD repeat-containing protein | RHS | 0.68 | 0.01 | 0.00 |
| BspSym | BAT02231 | Insecticidal toxin protein | RHS | 0.40 | 0.01 | 0.00 |
| BspSym | BAT02232 | Probable RHS | probable RHS | 0.51 | 0.01 | 0.00 |
| BspSym | BAT02232 | YD repeat-containing protein | RHS | 0.51 | 0.01 | 0.00 |
| BspSym | BAT02233 | YD repeat-containing protein | RHS | 0.40 | 0.01 | 0.00 |
| BspSym | BAT02234 | Insecticidal toxin complex protein tccc (Toxin complex protein) | RHS | 0.69 | 0.01 | 0.00 |
| BspSym | BAT02235 | Probable RHS | probable RHS | 0.44 | 0.00 | 0.01 |
| BspSym | BAT02238 | Probable RHS | probable RHS | 0.49 | 0.00 | 0.00 |
| BspSym | BAT02239 | Probable RHS | probable RHS | 0.39 | 0.01 | 0.00 |
| BspSym | BAT02240 | Probable RHS | probable RHS | 0.26 | 0.00 | 0.00 |
| BspSym | BAT02242 | Insecticidal toxin protein | RHS | 0.52 | 0.00 | 0.00 |
| BspSym | BAT02244 | Probable RHS | probable RHS | 0.26 | 0.00 | 0.00 |
| BspSym | BAT00289-290 | Rtxa | RTX | 0.53 | 0.01 | 0.00 |
| BspSym | BAT00052-EXT | RTX toxin | RTX | 0.39 | 0.01 | 0.00 |
| BspSym | BAT00290-end | Rtx | RTX | 0.33 | 0.01 | 0.01 |
| BspSym | BAT00101 | Probable RHS | probable RHS | 0.53 | 0.02 | 0.00 |
| BspSym | BAT01112 | Probable RHS | probable RHS | 1.10 | 0.00 | 0.02 |
| BspSym | BAT01459 | RTX toxin RtxA | MARTX | 0.48 | 0.02 | 0.03 |
| BspSym | BAT01453 | Hypothetical protein [MARTX] | MARTX | 0.46 | 0.01 | 0.01 |
| BspSym | BAT01127 | Probable RHS | probable RHS | 0.19 | 0.00 | 0.00 |
| BspSym | BAT01128 | Probable RHS | probable RHS | 0.35 | 0.00 | 0.00 |
| BspSym | BAT01129 | Probable RHS | probable RHS | 0.54 | 0.00 | 0.00 |
| BspSym | BAT01130 | Probable RHS | probable RHS | 0.31 | 0.00 | 0.00 |
| BspSym | BAT00154 | Hypothetical protein [MARTX] | MARTX | 0.51 | 0.01 | 0.00 |
| BspSym | BAT00160 | Hypothetical protein [MARTX] | MARTX | 0.51 | 0.00 | 0.00 |
| BspSym | BAT00158 | Hypothetical protein [MARTX] | MARTX | 0.43 | 0.00 | 0.00 |
| BspSym | BAT00156 | Hypothetical protein [MARTX] | MARTX | 0.41 | 0.00 | 0.00 |
| BspSym | BAT00152 | Martx | MARTX | 0.38 | 0.00 | 0.00 |
| BspSym | BAT00153 | Hemagglutination activity domain protein | MARTX | 0.29 | 0.00 | 0.00 |
| BspSym | BAT00159 | Hypothetical protein [MARTX] | MARTX | 0.27 | 0.01 | 0.00 |
| BspSym | BAT01461 | Hypothetical protein [MARTX] | MARTX | 0.14 | 0.00 | 0.00 |
| BspSym | BAT00161 | Hemolysin activator protein | MARTX activator | 0.25 | 0.00 | 0.00 |
| BspSym | BAT02224 | Probable RHS | probable RHS | 0.22 | 0.00 | 0.00 |
| BspSym | BAT01961 | TccC4 | RHS | 0.65 | 0.01 | 0.00 |
| BspSym | BAT00557 | Insecticidal toxin protein | RHS | 0.58 | 0.02 | 0.00 |
| BspSym | BAT02236 | Probable RHS | probable RHS | 0.38 | 0.01 | 0.01 |
| BspSym | BAT02243 | Probable RHS | probable RHS | 0.35 | 0.01 | 0.00 |
| BspSym | BAT02031 | Insecticidal toxin protein | RHS | 0.42 | 0.00 | 0.00 |
| BspSym | BAT00093 | Insecticidal toxin complex-like protein | RHS | 0.27 | 0.00 | 0.00 |
| BspSym | BAT00102 | Insecticidal toxin protein | RHS | 0.13 | 0.00 | 0.00 |
| BspSym | BAT00001 | Hemolysin-type calcium binding protein | RTX | 0.80 | 0.04 | 0.06 |
| BspSym | BAT00371 | Protein containing Type 1 secretion | RTX | 0.45 | 0.02 | 0.03 |
| BspSym | BAT00002 | RTX toxin protein | RTX | 0.22 | 0.02 | 0.02 |

^#^Rep = Replicate

Supplementary File 1E. Samples used in this study

| **Species** | **Cruise** | **Collection date** | **Site** | **Latitude** | **Longitude** | **Depth (m)** | **Sequencing center** | **Number of individuals** | **Purpose** | **Sequencing method** | **CO1 accession number** |
| --- | --- | --- | --- | --- | --- | --- | --- | --- | --- | --- | --- |
| *B*. sp. | Marsued 5 | 2 May 2009 | Lilliput | 09°32.82'S | 13°12.56'W | 1491 | Genoscope (France) | 1 | Genomics* | 454/  Illumina 36bp | - |
| *B*. sp. | M78-2 | 29 Apr 2009 | Lilliput | 09°32.85'S | 13°12.64'W | 1489 | Institute for Clinical Molecular Biology (Germany) | 3 | Transcriptomics | Illumina 100bp (PE^&^) | LN833438  LN833439  LN833440 |
| *B. azoricus* | RV Meteor cruise M82-3 | 10 Sep 2010 | Menez Gwen | 37°50.68’N | 31°31.17’W | 835 | Genoscope (France) | 1 | Genomics | 454 | LN833434 |
| *B. azoricus* | RV Meteor cruise M82-3 | 10 Sep 2010 | Menez Gwen | 37°50.68’N | 31°31.17’W | 835 | Genoscope (France) | 2 | Proteomics | - | - |
| *B. azoricus* | MoMARETO | 13 Aug 2006 | Menez Gwen | 37°45.58N | 31°38.26’W | 840 | OIST (Japan) | 1 | Genomics | 454 | LN833433 |
| *B. azoricus* | Biobaz | 9 Aug 2013 | Lucky Strike | 37°16.98”N | 32°16.54’W | 1700 | Max Planck Genome Centre (Germany) | 3 | Transcriptomics | Illumina 100bp | LN833435  LN833436  LN833437 |

*Longest scaffold published in Petersen et al. 2011

^#^Single host individuals were used for sequencing.

CO1 = cytochrome c oxidase subunit 1

^&^PE = Paired-end sequencing

Supplementary File 1F. Primer sequences and annealing temperatures used to detect genome rearrangements.

| **Primer pair** | **Forward** | **Reverse** | **Temp (°C)** | **Product size** |
| --- | --- | --- | --- | --- |
| BT1 | TCATGCGCTGACTCGCCATT | TCGTTCTATGCAAGTGGCACGCT | 62 | 1746* |
| BT4 | TGCCCCAAATCCCAAGGTGC | TTGATGGATCCTCCTGCAATGGCT | 62 | 1074* |
| BT5 | TCCAGTCTGGCGCTCACAGG | TGCTGCTGACGCTTTTGGACAG | 62 | 959 |
| BT6 | GGCCTAGCCCTATTTAGCAACCGGA | GCAAGTGCGTCAAAATCCACCGA | 65 | 1199* |

*The PCR product was of the expected size but not sequenced.

Supplementary File 1G. Metagenomes and metatranscriptomes enriched in SUP05 from oxygen minimum zones (OMZ) or hydrothermal vents.

| **Metagenome/ metatranscriptome** | **Accession number/Database** | **Class** | **Query** | **Best hit** | **E-value** | **% Identity** | **% Coverage** |
| --- | --- | --- | --- | --- | --- | --- | --- |
| Juan de Fuca chimney (metagenome) | SRX012322 | - | - | - | - | - | - |
| Lost City chimney biofilm (metagenome) | CAM_PROJ_Hydro-thermal Vent (CAMERA) | - | - | - | - | - | - |
| Lost City chimney (metagenome) | ACQI00000000 | YD | BAT02242 | ACQI01008516 | 7.00E-08 | 31 | 75 |
| Guaymas basin plume (metagenome/metatranscriptome) | AJXC00000000 | RTX | BAT00001 | GB_4MN_MetaGALL_nosff_rep_c209590 | 6.85E-06 | 34 | 64 |
| OMZ of East tropical South Pacific Chile (metagenome) | SRA025088 | - | - | - | - | - | - |
| OMZ of East tropical South Pacific Chile (metagenome/metatranscriptome) | SRA023632 | - | - | - | - | - | - |

Supplementary File 1H. Amino acid sequences from the following genomes were used in the reference database for proteomic analysis (IncDB). The genomes belong to relatives of the sulfur-oxidizing (SOX) and methane-oxidizing (MOX) symbionts of *Bathymodiolus azoricus*, as well as the mussel host.

|  | **Sequences of *Bathymodiolus*** | **Sequences of phylogenetic relatives** | |
| --- | --- | --- | --- |
|  |  | **Symbiotic (*gamma-proteobacteria*)** | ***Free-living (*gamma-proteobacteria*)** |
| **SOX** | SOX sequences from available *Bathymodiolus azoricus* EST library (Bettencourt et al., 2010)  SOX ORFs from draft genomes of *Bathymodiolus azoricus* (This study: BazSymA and BazSymB)  SOX ORFs from draft genome of *Bathymodiolus sp.* from Lilliput, in the SouthMAR (This study: BspSym) | *Olavius algarvensis Gamma1* and *Gamma3 symbionts* (Kleiner et al., 2012)  *Ca.* Endoriftia persephone and endosymbiont of *Tevnia jerichonana* (Gardebrecht et al., 2012)  *Ca.* Ruthia magnifica str. Cm (Newton et al., 2007)  *Ca.* Vesicomyosocius okutanii HA (Kuwahara et al., 2007) | *Acidithiobacillus ferrooxidans, Allochromatium vinosum DSM 180, Beggiatoa sp. SS, Beggiatoa sp. PS, Beggiatoa alba B18LD Caminibacter mediatlanticus TB-2, Halothiobacillus neapolitanus c2, ARCTIC020, ARCTICb15, SUP05, Magnetococcus marinus MC-1, Marichromatium purpuratum 984, Nitrosococcus halophilus Nc4, Nitrosococcus oceani ATCC 19707, Nitrosococcus watsonii C-113, Paracoccus denitrificans PD1222, Persephonella marina EX-H1, Starkeya novella* DSM 506, *Sulfurimonas gotlandica GD1, Sulfurimonas denitrificans* DSM 1251, *Sulfurimonas autotrophica* DSM 16294, *Thermovibrio ammonificans HB-1,* DSM 15698*, Thiobacillus denitrificans* ATCC 25259*, Thiocapsa marina* 5811*, Thiocystis violascens* DSM 198, *Thiomicrospira crunogena* XCL-2, *Thiorhodococcus drewsii* AZ1*, Thiorhodovibrio* sp. 970 |
| **MOX** | MOX sequences from available *Bathymodiolus azoricus* EST library  MOX ORFs from draft genome of *Bathymodiolus azoricus* (BazSymA)  MOX ORFs from draft genome of *Bathymodiolus sp.* from SouthMAR (BspSym) | - | *Methylobacter tundripaludum SV96, Methylococcus capsulatus str. Bath, Methylomicrobium alcaliphilum*, *Methylomonas methanica* MC09, all amino acid sequences available in NCBI under phylogenetic group *Methylococcaceae* |
| **Host** | Host sequences from available *Bathymodiolus azoricus* EST library with bacterial sequences removed. | *Mytilus edulis, Mytilus galloprovincialis, Mizuhopecten yessoensis,* all amino acid sequences available in NCBI under phylogenetic group Bivalvia. | |

*These sequences were downloaded from available NCBI genome BioProjects.

**Supplementary File 1I. Details of expressed toxin-related proteins identified with proteomics. The values are given in % NSAF, which is a normalized spectral abundance factor that gives the relative abundance of a protein in a sample in %.**

| **Identifier** | **Category** | **Max. coverage (%)^#^** | **Gill A** | **Gill B** | **Foot A** | **Foot B** | **Sup. A** | **Sup. B** | **GP A** | **GP B** | **MGP** | **MGill** |
| --- | --- | --- | --- | --- | --- | --- | --- | --- | --- | --- | --- | --- |
| Host_EST_000107 | YD | 33 | 0.0082 | 0.0234 | 0.0000 | 0.0000 | 0.0040 | 0.0055 | 0.0188 | 0.0628 | 0.0318 | 0.0115 |
| Host_EST_000115 | YD | 34 | 0.0117 | 0.0319 | 0.0000 | 0.0000 | 0.0041 | 0.0113 | 0.0299 | 0.0814 | 0.0325 | 0.0265 |
| Host_EST_000248 | YD | 33 | 0.0019 | 0.0204 | 0.0000 | 0.0000 | 0.0000 | 0.0064 | 0.0170 | 0.0389 | 0.0000 | 0.0000 |
| Host_EST_002123 | YD | 24 | 0.0000 | 0.0000 | 0.0000 | 0.0000 | 0.0000 | 0.0000 | 0.0000 | 0.0000 | 0.0142 | 0.0361 |
| Thio_BAZ_1943_contig360420_0 | RTX (activator) | 21 | 0.0037 | 0.0044 | 0.0000 | 0.0000 | 0.0090 | 0.0125 | 0.0189 | 0.0000 | 0.0000 | 0.0130 |
| Tox_BAZ_119_contig00027_0 | YD | 18 | 0.0054 | 0.0035 | 0.0000 | 0.0000 | 0.0005 | 0.0000 | 0.0210 | 0.0333 | 0.0000 | 0.0086 |
| Tox_BAZ_120_contig00027_1 | YD | 27 | 0.0215 | 0.0284 | 0.0027 | 0.0000 | 0.0028 | 0.0019 | 0.0246 | 0.0653 | 0.0358 | 0.0420 |
| Tox_BAZ_1734_contig02141_2 | RTX (transporter) | 46 | 0.0063 | 0.0024 | 0.0137 | 0.0000 | 0.0000 | 0.0000 | 0.0055 | 0.0287 | 0.0556 | 0.0317 |
| Tox_BAZ_2494_contig00030_0 | YD | 29 | 0.0119 | 0.0116 | 0.0006 | 0.0000 | 0.0017 | 0.0007 | 0.0077 | 0.0257 | 0.0551 | 0.0582 |
| Tox_BAZ_3202_scaffold00038_7 | RTX | 8.7 | 0.0000 | 0.0000 | 0.0000 | 0.0000 | 0.0000 | 0.0000 | 0.0000 | 0.0000 | 0.0098 | 0.0071 |
| Tox_BAZ_525_contig104979_0 | YD | 6.8 | 0.0000 | 0.0016 | 0.0000 | 0.0000 | 0.0000 | 0.0000 | 0.0017 | 0.0069 | 0.0000 | 0.0024 |
| ToxAzor_892893 | YD | 2.8 | 0.0009 | 0.0020 | 0.0000 | 0.0000 | 0.0000 | 0.0000 | 0.0000 | 0.0011 | 0.0000 | 0.0000 |
| ToxSMAR_1260BAT01109 | YD | 3.4 | 0.0020 | 0.0069 | 0.0000 | 0.0000 | 0.0007 | 0.0000 | 0.0015 | 0.0115 | 0.0080 | 0.0081 |
| ToxSMAR_2052BAT01788, Thio_BAZ_1733_contig02141_1 or Thio_BAZ_2580_scaffold00010_8 | RTX (transporter) | 8 | 0.0023 | 0.0038 | 0.0000 | 0.0000 | 0.0000 | 0.0000 | 0.0045 | 0.0184 | 0.0000 | 0.0000 |
| ToxSMAR893-894 | YD | 1.8 | 0.0000 | 0.0008 | 0.0000 | 0.0000 | 0.0000 | 0.0000 | 0.0000 | 0.0017 | 0.0000 | 0.0000 |
| ToxAzor_890891 | YD | 9.9 | 0.0041 | 0.0076 | 0.0000 | 0.0000 | 0.0016 | 0.0000 | 0.0088 | 0.0284 | 0.0153 | 0.0148 |

Sup. = supernatant; GP = Gradient Pellet; MGP = Membrane proteome of the gradient pellet; MGill = Membrane proteome of the gill.

^#^The highest coverage of the protein sequence by identified peptides in one sample.

Bettencourt, R., Pinheiro, M., Egas, C., Gomes, P., Afonso, M., Shank, T., and Santos, R.S. (2010). High-throughput sequencing and analysis of the gill tissue transcriptome from the deep-sea hydrothermal vent mussel *Bathymodiolus azoricus*. BMC Genomics *11*, 559.

Gardebrecht, A., Markert, S., Sievert, S.M., Felbeck, H., Thürmer, A., Albrecht, D., Wollherr, A., Kabisch, J., Bris, N.L., Lehmann, R., et al. (2012). Physiological homogeneity among the endosymbionts of *Riftia pachyptila* and *Tevnia jerichonana* revealed by proteogenomics. ISME J. *6*, 766–776.

Kleiner, M., Petersen, J.M., and Dubilier, N. (2012). Convergent and divergent evolution of metabolism in sulfur-oxidizing symbionts and the role of horizontal gene transfer. Curr. Opin. Microbiol. *15*, 621–631.

Kuwahara, H., Yoshida, T., Takaki, Y., Shimamura, S., Nishi, S., Harada, M., Matsuyama, K., Takishita, K., Kawato, M., Uematsu, K., et al. (2007). Reduced genome of the thioautotrophic intracellular symbiont in a deep-sea clam, *Calyptogena okutanii*. Curr. Biol. *17*, 881–886.

Newton, I.L.G., Woyke, T., Auchtung, T.A., Dilly, G.F., Dutton, R.J., Fisher, M.C., Fontanez, K.M., Lau, E., Stewart, F.J., Richardson, P.M., et al. (2007). The *Calyptogena magnifica* Chemoautotrophic Symbiont Genome. Science *315*, 998–1000.

Walsh, D.A., Zaikova, E., Howes, C.G., Song, Y.C., Wright, J.J., Tringe, S.G., Tortell, P.D., and Hallam, S.J. (2009). Metagenome of a versatile chemolithoautotroph from expanding oceanic dead zones. Science *326*, 578–582.
